# Supplementary figures and images for: KFERQ-selective protein autophagy in Caenorhabditis elegans depends on LMP-1
Source: PLoS One. 2025 Sep 2;20(9):e0330339. doi: 10.1371/journal.pone.0330339 (PMC12404552; doi:10.1371/journal.pone.0330339)

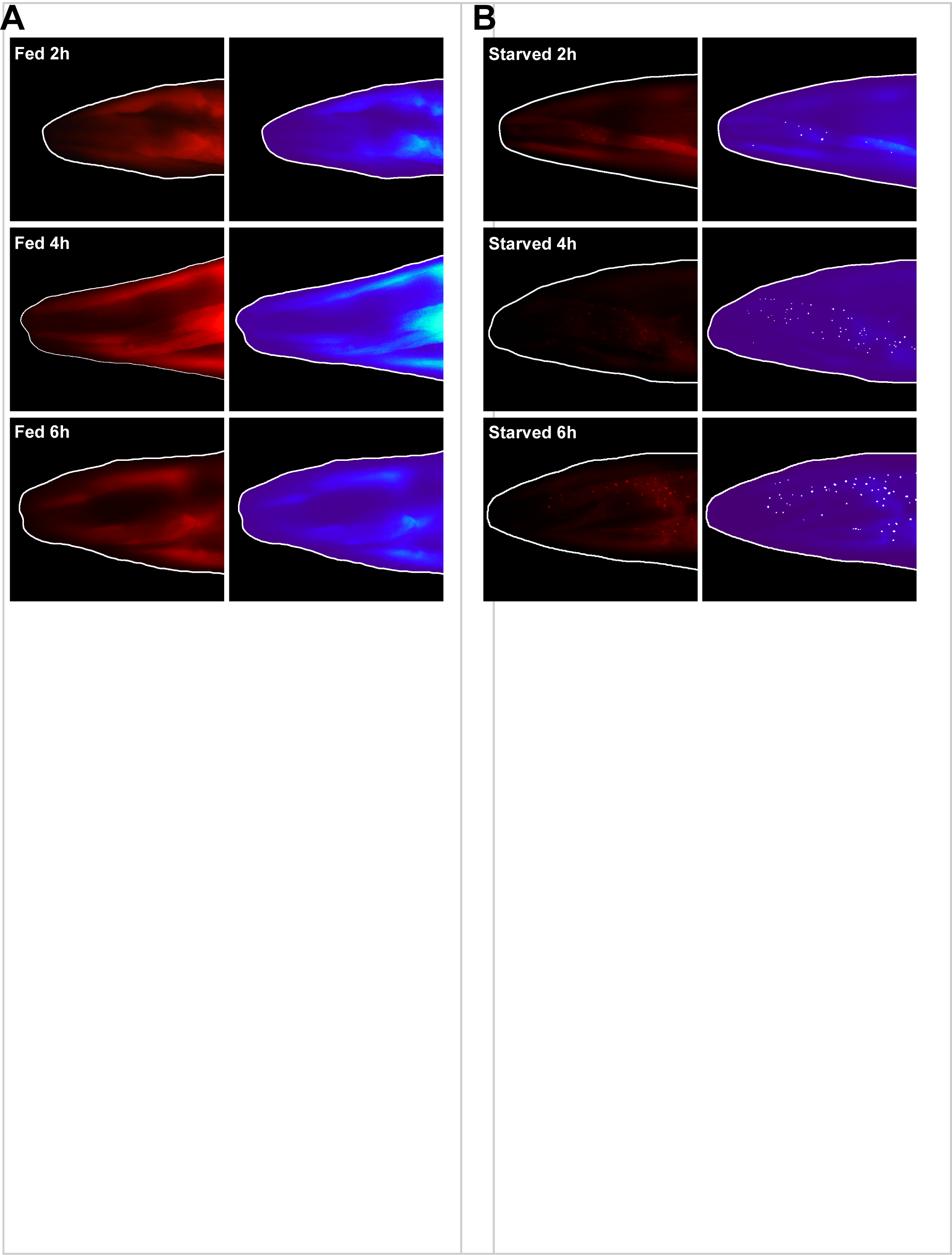

Supplement: S1 Fig — (A) Representative image of fed worms (corresponding to Fig 1C, panel a). (B) Representative image of starved worms (corresponding to Fig 1C, panels b–d). Masks were created by thresholding and particle analysis in ImageJ to quantify puncta associated with the KFERQ reporter. All images were processed under the same parameters for accurate comparison. (TIF) [file pone.0330339.s001.tif]

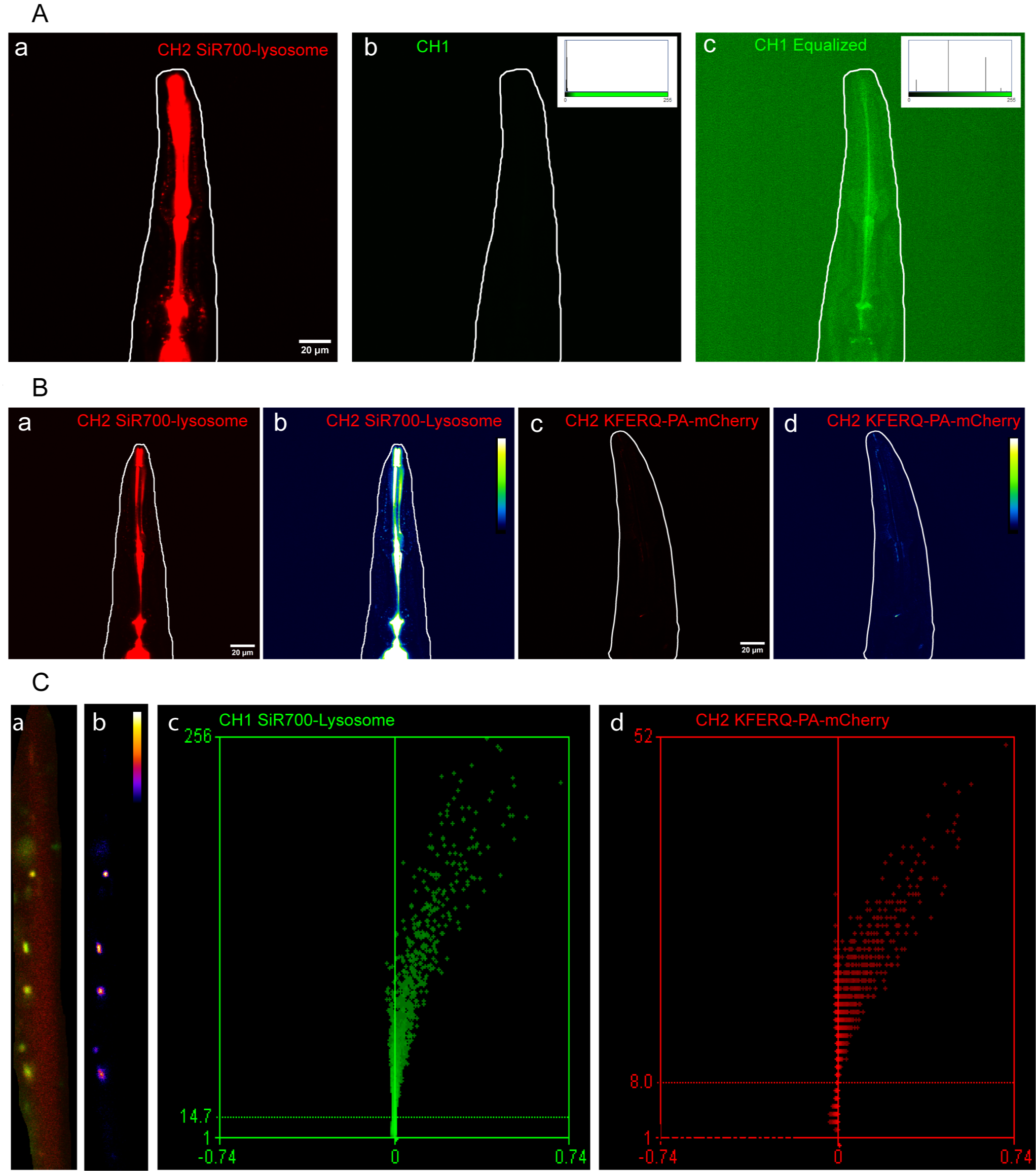

Supplement: S2 Fig — a) Confocal image of a worm head stained with the Sir700-lysosome probe (CH2; excitation: 635 nm; emission: 655–755). b) Confocal image of a worm head from a non-photoactivated animal stained with SiR700-lysosome probe observed in the mCherry channel (CH1: excitation: 543 nm; emission: 572 ± 20 nm). c) The image in b with enhanced contrast using histogram equalization to show signal presence in b. Scale bar = 20 μm. B. Comparison of the signal intensity in Sir700-lysosome fluorescence channel (lysosomal probe) vs. KFERQ-PAmCherry fluorescence. a) Image of a worm’s head stained with SiR700-lysosome (CH2; excitation: 635 nm; emission: 655–755) and b) corresponding pseudocolored image. c) Image of a photoactivated worm expressing KFERQ-PA-mCherry observed in the Sir700 channel (CH2; excitation: 635 nm; emission: 655–755), and d) corresponding pseudocolored image acquired using the same settings as in a and b. C. Intensity correlation analysis of KFERQ-PAmCherry with the lysosomal activity marker SiR700-lysosome. a) Representative image of muscle tissue from the head region showing the KFERQ-PAmCherry reporter (red) following photoactivation and incubation with SiR700- lysosome (green). Yellow puncta show areas of colocalization between the signals from both channels, indicating overlap between KFERQ-PAmCherry and lysosomes. b) Pseudocolored image showing the correlation of signal intensities between KFERQ-PAmCherry and SiR700-Lysosome. Plots c and d show the degree of synchronization of the KFERQ-PAmCherry and SiR700-Lysosome signals (products of the differences of the intensity of each pixel with respect to the mean intensity of each channel) normalized with respect to the SiR700-Lysosome and PAmCherry signal. (TIF) [file pone.0330339.s002.tif]

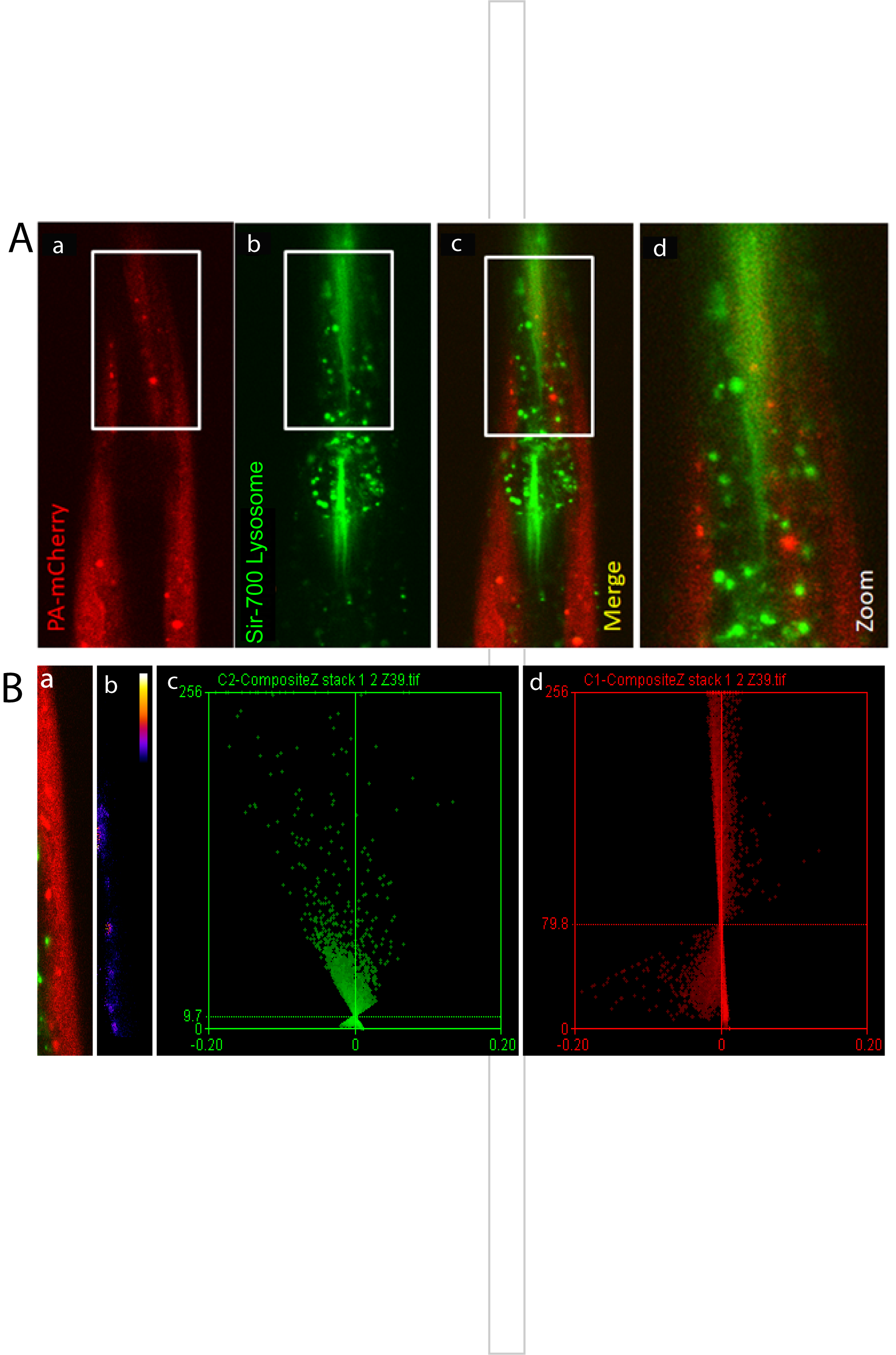

Supplement: S3 Fig — A) Representative confocal images of 2-day old transgenic adult worm of expressing PAmCherry in muscle cells. mCherry (red 572/50 emission) and the Lys700 lysosomal SiR700-lysosome (green). (a) PAmCherry reporter fluorescence in head muscles (b) SiR700-lysosome fluorescence image (green). (c) Merge of the images in a and b, yellow indicates colocalization of the mCherry reporter with lysosomes. (d) Zoom of the region labeled in (a), (b) and (c). B) Intensity correlation analysis of PAmCherry with lysosomal activity. (a) Representative image of nematode head muscle tissue in which the PAmCherry reporter (red) was photoactivated and incubated with SiR700-lysosome (green). (b) Pseudocolored image showing the correlation of signal intensities between PAmCherry and SiR700-lysosome. Plots (c) and (d) show the degree of synchronization of the PAmCherry and SiR700-lysosome signals (products of the differences of the intensity of each pixel with respect to the mean intensity of each channel) normalized with respect to the SiR700-lysosome (c) and PAmCherry (d) signal. (delta KFERQ). (TIF) [file pone.0330339.s003.tif]

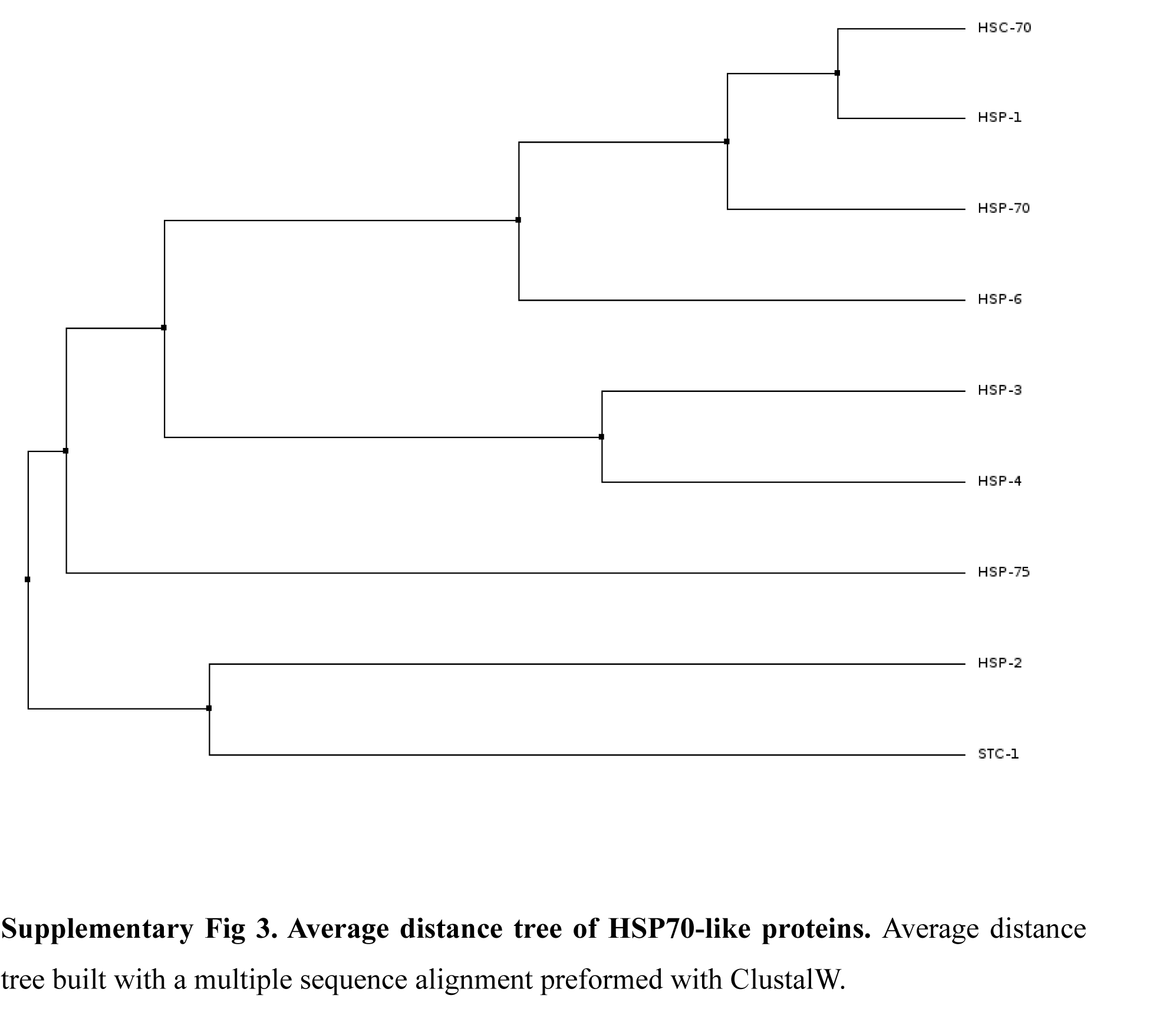

Supplement: S4 Fig — Average distance tree built with a multiple sequence alignment preformed with ClustalW. (TIF) [file pone.0330339.s004.tif]

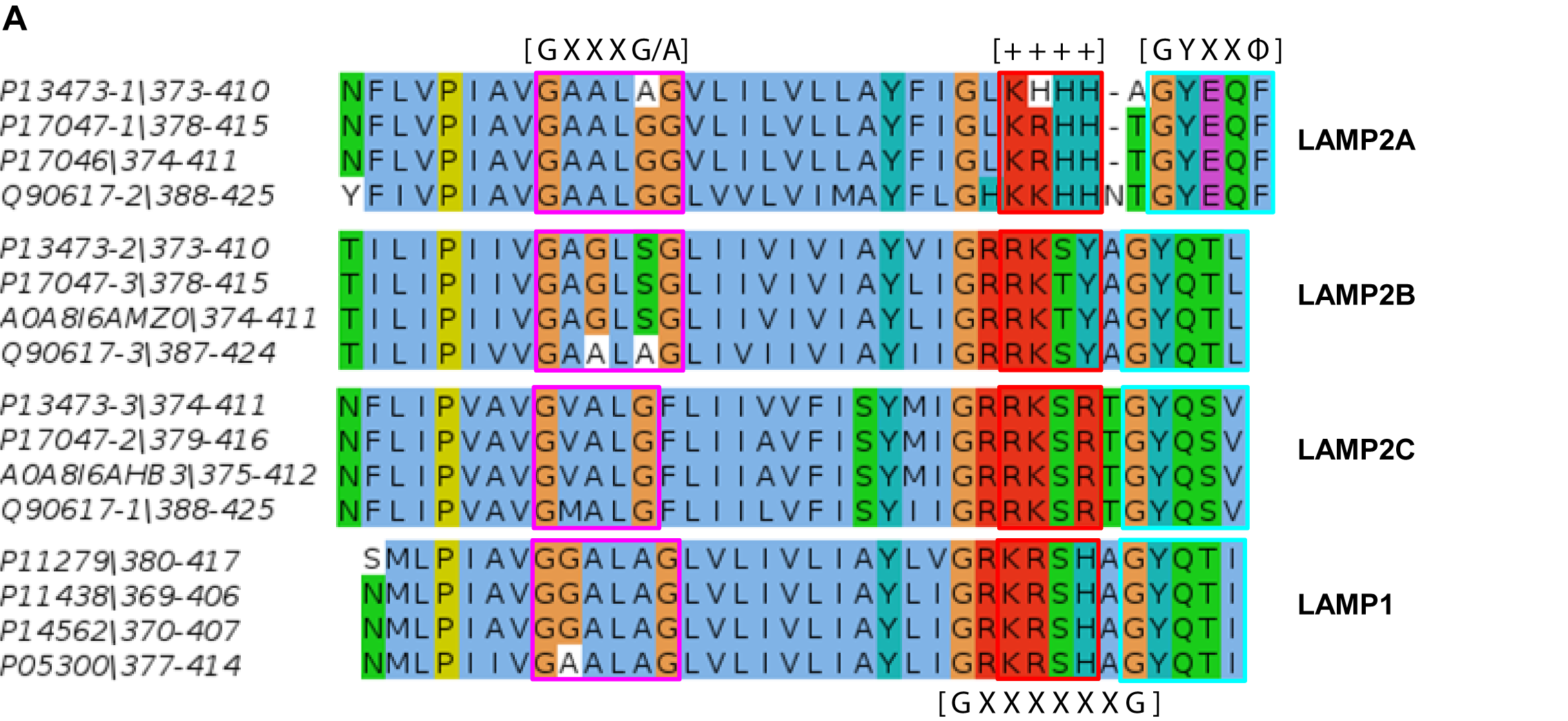

Supplement: S5 Fig — All LAMP2 isoforms and LAMP1 are following the same order (human, mouse, rat and chicken). Name on the right is based on the nomenclature used for human sequences. From left to right (N- to C-terminal) the motif for transmembrane homotypic interactions [GXXXG/A], the C-terminal cytosolic motif where important residues for LAMP2A/HSC70 are found [GXXXXXXXG], and the C-terminal end lysosome destination signal [GYXXΦ], are indicated. Positive residues in the [GXXXXXXXG] domain is shown in red. (TIF) [file pone.0330339.s005.tif]
