# Supplementary material for: KFERQ-selective protein autophagy in Caenorhabditis elegans depends on LMP-1
Source: PLoS One. 2025 Sep 2;20(9):e0330339. doi: 10.1371/journal.pone.0330339 (PMC12404552; doi:10.1371/journal.pone.0330339)
Supplement: Attachment — Submitted filename: Answer to reviewers May.pdf [file pone.0330339.s010.pdf]

Comments to the Author

1. If the authors have adequately addressed your comments raised in a previous round of review and you feel that this manuscript is now acceptable for publication, you may indicate that here to bypass the "Comments to the Author" section, enter your conflict-of-interest statement in the "Confidential to Editor" section, and submit your "Accept" recommendation.

Reviewer #1: (No Response)

Reviewer #2: (No Response)2. Is the manuscript technically sound, and do the data support the conclusions?

The manuscript must describe a technically sound piece of scientific research with data that supports the conclusions. Experiments must have been conducted rigorously, with appropriate controls, replication, and sample sizes. The conclusions must be drawn appropriately based on the data presented.

Reviewer #1: Partly

Reviewer #2: Partly

*We understand that the main problem is that high-resolution images are not available to the reviewers in eps or tiff format, and therefore they are of very low quality. The journal process for submission only allows figures to be uploaded with a maximum of 10 Mb, but we were able to upload them in a quality sufficient enough for their analyses. However, when the final version is converted into pdf in the journal's platform, the quality of the image's decreases, so we agree with the reviewers that they are difficult to visualize. They must actively search for them in the repository where we include graph data and images in EPS and TIFF format as has been requested (**Harvard dataverse " https://doi.org/10.7910/DVN/DFPZQX**". The reviewers can download all images as well as raw graph data.*

3. Has the statistical analysis been performed appropriately and rigorously?

Reviewer #1: I Don't Know

Reviewer #2: Yes

*The numerical data (graphs and raw data) and statistical analysis have been uploaded into Harvard database repository **https://doi.org/10.7910/DVN/DFPZQX***

Moreover, in every figure legend we specified the statistical tests used, the sample size, and the number of independent assays.

4. Have the authors made all data underlying the findings in their manuscript fully available?

The requires authors to make all data underlying the findings described in their manuscript fully available without restriction, with rare exception (please refer to the Data Availability Statement in the manuscript PDF file). The data should be provided as part of the manuscript or its supporting information, or deposited to a public repository. For example, in addition to summary statistics, the data points behind means, medians and variance measures should be available. If there are restrictions on publicly sharing data—e.g. participant privacy or use of data from a third party—those must be specified.

Reviewer #1: No

Reviewer #2: No

*All numerical data (graphs and raw data) has been uploaded into Harvard database repository **https://doi.org/10.7910/DVN/DFPZQX** This link was already available in the answer to the reviewers (point 3) and in the pdf created in the journal´s platform and in "data availability".*

5. Is the manuscript presented in an intelligible fashion and written in standard English?

PLOS ONE does not copyedit accepted manuscripts, so the language in submitted articles must be clear, correct, and unambiguous. Any typographical or grammatical errors should be corrected at revision, so please note any specific errors here.

Reviewer #1: Yes

Reviewer #2: Yes

6. Review Comments to the Author

Please use the space provided to explain your answers to the questions above. You may also include additional comments for the author, including concerns about dual publication, research ethics, or publication ethics. (Please upload your review as an attachment if it exceeds 20,000 characters)

Reviewer #1: The authors have responded to all reviewers' comments and have defended several of them. However, some issues remain including major ones.

Major

1. The quality of the microscopic images remains low. I do not have access to .eps files and cannot judge the content.

We understand that the main problem is that high-resolution images are not available to the reviewers in eps or tiff format, and therefore they are of very low quality. The journal process for submission only allows figures to be uploaded with a maximum of 10 Mb, but we were able to upload them in a quality sufficient for their analyses. However, when the final version is converted into pdf in the journal's platform, the quality of the images decreases in such a way, so we agree with the reviewers that they are difficult to visualize. Unfortunately, they must actively search for them in the repository where we include graph data and images in EPS and TIFF format as has been requested (Harvard dataverse ***https://doi.org/10.7910/DVN/DFPZQX*****).** *All data can be downloaded directly from this platform.*

2. The issue concerning puncta quantification is not only to describe the analysis procedure. To my eyes some images cannot be easily subjected to quantification. The masks of quantified particles can answer the above.

We expect that this observation would be answered satisfactorily if the images could be seen in higher quality. Despite this, we included a supplementary (S1) figure with the masks of quantified particles of a set of representative pictures from Fig1C .

It is important to be aware that the expression of KFERQ-PAmCherry is mainly cytosolic and that upon starvation it acquires a punctate distribution. However, some cytosolic fluorescence always remains.

3. Indeed, the head muscles are part of the body wall musculature. However, the comment was referring to the thrashing assay and the impact of the rest of the body wall muscle cells in the thrashing assay. Those cells outnumber the head muscle cells and they are at least equally well-defined anatomically.

The body wall muscles are one tissue, from head to tail. What happens in the head muscles also occurs in the muscles of the rest of the body. Thrashing is an assay that is used to evaluate mobility, which is affected when there is muscle or neuronal defects. In particular, the effects of alpha-synuclein aggregates in muscle cells of this strain have been characterized using this assay. (doi: 10.3390/ph15050512). The autofluorescence of the intestine and presence of eggs in the uterus make the visualization of alpha-synuclein aggregates in body wall muscle cells very difficult and unreliable, except for the head region.

Minor

4. My title suggestion referred to the grammar. A “destination” cannot be “triggered” or “increased”.

The reviewer is correct. We have changed the sentence to: “Starvation promotes KFERQ-PAmCherry delivery to lysosomes in C. elegans muscle cells”.

5. The word “dots” still remains in some cases.

The remaining “dots” words have been changed.

6. Fig. 1D legend: In the graph, there are three and not four asterisks.

The missing asterisk has been added in the graph.

7. Sup. Fig. 1B and Sup. Fig. 2Bb: Heatmaps are still missing.

Heatmaps were added. Sup. Fig 2Bb is now S3Bb.

8. Reference 59 appears before reference 54.

Reference #59 was corrected and incorporated in line 451 (new manuscript).

Reviewer #2: The authors have revised the manuscript.

First a general comment: The authors should provide the response to the reviewers beneath the respective questions. This will make the reviewing easier.

Specifically, the authors have provided images in which the puncta/aggregates could be seen. This was the major concern as the whole study was based on this precinct. Now, the manuscript is a lot better. But the following concerns need to be addressed.

1. In figure 1C-d, KFERQ- mCherry puncta upon starvation could be seen. The lysosomal targeting image is provided. More than the number of puncta (Fig. 1D), the number of puncta localized to the lysosome could be a better indicator of chaperone induced autophagy (CIA). This needs to be included. Further, this will reduce the reliance on the subjective counting of aggregates with varying sizes and shapes that the authors have provided as an explanation to Reviewer 1's query.

The results in Figure 1 E show that almost 100% of the KFERQ-PAmCherry puncta co-localize with the lysosomal marker in muscle cells. The confocal images in Fig 1E also show that the synchronization of the lysosomal signal (Sir700-lysosome) increases with that of the positive KFERQ-PAmCherry particles, occurring in all cases (Fig E, e-h). However, there are few lysosomal particles that do not co-locate with KFERQ (Fig E, f-g, arrowhead), either because they are not positive for CMA or because they are found in muscle-adjacent tissues (pharynx, hypodermis, and neurons) that do not express the fluorescent reporter.

Besides the analysis we performed of image in Fig. 1E, we analyzed several other confocal images finding the same results (images below). First, we identified the KFERQ-PAmCherry expressing tissue (red), then we counted the puncta. We found that in contrast to the results with PAmCherry overexpression in Supplementary Fig S3*, almost 100% KFERQ-PAmCherry puncta colocalized with Sir700-lysosome in head muscle cells, with only a few puncta of smaller size per image being exclusively Sir700-lysosome positive (and we cannot be sure they are in other tissues). All the other green puncta are in tissues that do not express KFERQ-PAmCherry and therefore are not body wall muscle tissue.*

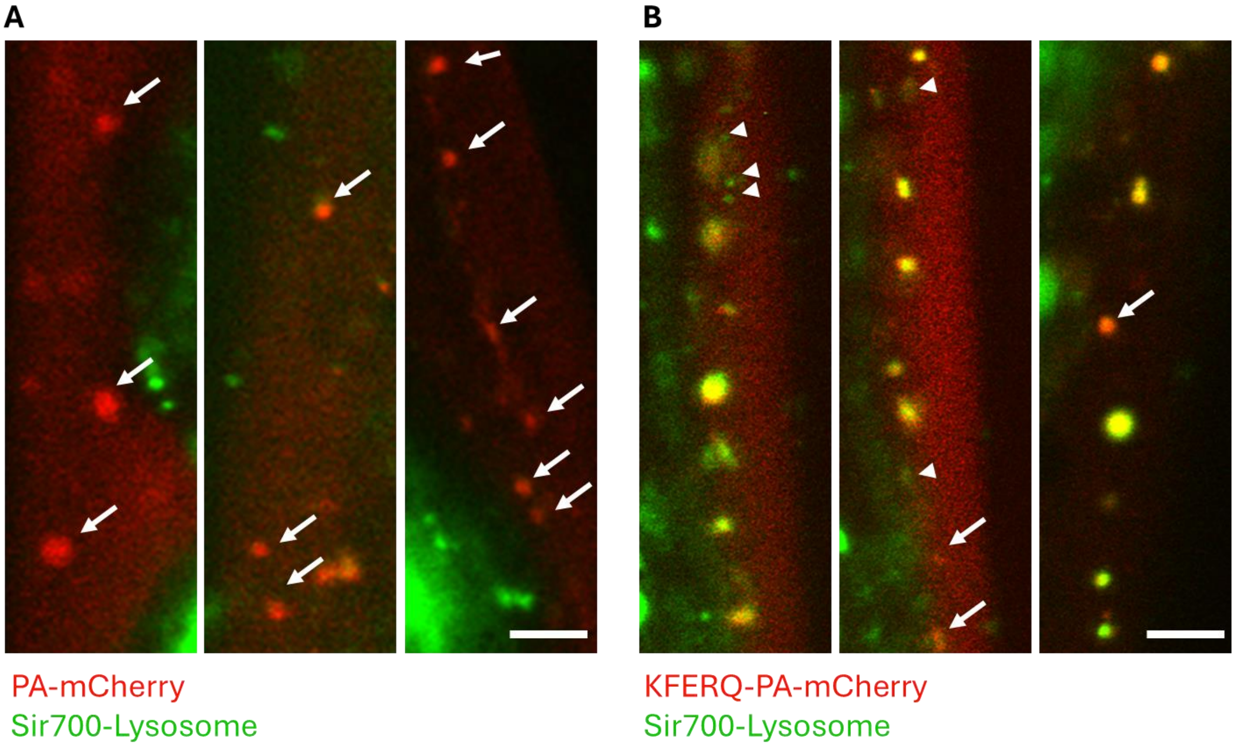

Fig 1. Confocal microscopy images of C. elegans head muscles expressing the CMA reporter, in which colocalization analysis was performed between A) PA-mCherry (deleted KFERQ) and Sir700-lysosome and B) KFERQ-PA-mCherry- and Sir700-lysosome marker (ICA, corresponding to Supplementary Figure 1C and S3).The total number of fluorescent spots in the muscle region that showed colocalization, the number of spots labeled with the lysosomal tracer alone and the fluorescent spots in the PA-mCherry channel were quantified. The results indicated that only 20% of lysosomes present in muscle cells did not colocalize with the CMA marker (arrowheads). In contrast, less than 0.1 % of the total number of fluorescents on average corresponded to KFERQ-PA-mcherry- that did not colocalize with the lysosomal activity marker (Fig 1B, arrows). (Scale bar = 5 µm)

2. In Fig. 3, lgg-1 RNAi results in increase in puncta number. Does this indicate activation of CIA? A double RNAi for lgg-1 and lmp-1 can clarify this to state that a GABARAP-independent autophagy is contributing to this. This will add strength to lmp-1 role in CIA in C. elegans.
Addition of this data will be ideal. Otherwise, include the potential additional role of lmp-1 in sentence 311.

We thank the reviewer for the insightful and constructive comments, which have helped improve the clarity and quality of our manuscript. The reviewer is right. It is possible that the phenotype of the double RNAi for lgg-1 and lmp-1 could add strength to lmp-1 role in CIA in C. elegans.

To address this, as the reviewer suggestion w–e included in sentence 311 "Additionally, in the absence of macroautophagy, lmp-1 expression can increase, and its stabilization at the lysosomal membrane could be enhanced to support increased CMA flux [1]."

3. In, Fig.4, alpha-synuclein aggregates increase in lmp-1 RNAi and the aggregate number reduce upon starvation which is the opposite of what is seen with KFERQ-PAmCherry(Fig.1) and lmp-1RNAi/mutant(Fig.2). Hence, is there any involvement of lmp-1 mediated CIA? Does LMP-1 reduce alpha- synuclein by any other mechanism? A lysosomal co-staining would have helped here to show lmp-1 involvement in autophagy and lysosomal targeting or another mechanism.

Fig. 4A does not show that the alpha-synuclein aggregates increase in lmp-1 RNAi. In fact, it shows that the absence of lmp-1 under starvation conditions does not decrease the number of aggregates as it happens when lmp-1 is present. It has been shown that CMA is able to deliver alpha-synuclein monomers to the lysosome, but not the aggregates (J Clin Invest. 2008 Feb;118(2):777-88. doi: 10.1172/JCI32806). Therefore, our evidence suggests that the decrease in the total number of aggregates is a consequence of this process of decreased number of monomers available for aggregation. We do not expect that CMA influences the process of aggregation directly.

4. Both Fig.3 and Fig.4, lgg-1 RNAi increasing KFERQ-PAmCherry puncta (Fig.3), and lmp-1 RNAi increasing alpha-synuclein aggregates(fig.4) indicate an overlap and/or some other function of lmp-1. The authors can include this in the discussion.

The answer to this query is the same as for the reviewer's comment 3.

5. In Discussion sentence 444, for the usage of "mature lysosome" is there any evidence provided? Otherwise, change it to "lysosomes".

The word "mature" was deleted.

7. PLOS authors have the option to publish the peer review history of their article (what does this mean?). If published, this will include your full peer review and any attached files.

If you choose "no", your identity will remain anonymous but your review may still be made

public.

Do you want your identity to be public for this peer review? For information about this choice, including consent withdrawal, please see our Privacy Policy.

Reviewer #1: No

Reviewer #2: No
