# Supplementary material for: KFERQ-selective protein autophagy in Caenorhabditis elegans depends on LMP-1
Source: PLoS One. 2025 Sep 2;20(9):e0330339. doi: 10.1371/journal.pone.0330339 (PMC12404552; doi:10.1371/journal.pone.0330339)
Supplement: Attachment — Submitted filename: Response to Reviewers.docx [file pone.0330339.s009.docx]

Answer to Editor:

First, we would like to sincerely thank the reviewers for their insightful feedback and valuable contributions to this work. Their precise observations have significantly improved the clarity and quality of the manuscript.

Journal requirements:

1. Files were uploaded with the required file names.
2. We fixed the information and provided grant number in the Funding Information section.
3. We added the required statement below.
4. Find below the amended Funding Statement as detailed in the guide for authors, and added the required statement as follows:

National Agency for Research and Development (ANID), https://anid.cl/: Financiamiento Basal para Centros Científicos y Tecnológicos de Excelencia #FB210008 to IEA  and CFL, FONDECYT#11161056 to IEA, and FONDECYT Posdoctorado 2021- #3210596 to GN. This research was partially supported by the supercomputing infrastructure of the NLHPC (#CCSS210001), https://www.nlhpc.cl/. Some strains were provided by the CGC https://cgc.umn.edu/, which is funded by NIH Office of Research Infrastructure Programs (P40 OD010440).

There was no additional external funding received for this study. The funders had no role in study design, data collection and analysis, decision to publish, or preparation of the manuscript.

5. We included a separate caption for each figure in the manuscript.
6. The supplementary figures´ file types were changed to “Supporting Information”
7. We did not use LaTeX to create our manuscript.

Answer to the reviewers’ comments:

2. The statistical test used and the sample size for each experiment are indicated in the figure legends.

3. Data was uploaded to https://dataverse.harvard.edu/dataset.xhtml?persistentId=doi:10.7910/DVN/DFPZQX.

Review comments to the author

Reviewer #1

Major comments:

1 y 2. The figures were originally submitted in pdf format and were therefore of very low quality. We have now submitted the original .eps files.

With respect to the possible overexposure of some photographs: Figure 1C: all photographs were taken with the same magnification and exposure, which allowed us to detect the distribution pattern of the fluorescent marker using an epifluorescence microscope (not confocal). With this exposure we can show that the florescent marker is localized in the cytosol under fed conditions, while under starvation conditions the pattern shifts to a more punctuated pattern over time. In the new version, this pattern can be seen more clearly, especially if the figure is zoomed in. This explanation is also valid for figures 2A, 2F and 3A.

3. All relevant information about the protocol for KFERQ-PAmCherry punta number measurement was added to the Material and Methods section as follows (from line 214-221): briefly, Image analyses of the puncta number in the worms’ head region were performed using the ImageJ software. The images were converted into 8-bit format, contrast was enhanced, and KFERQ-PAmCherry puncta were counted using the software cell counter tool. For figure 4D, showing an example of how the α-synuclein aggregates (of different sizes) were quantified. The answer to the question: that is effectively what we counted; all aggregates found in the head region of the worm. The aggregates that we can identify have different shapes and sizes, and some that we count as one aggregate could be 2 aggregates that are very close together.

4. Reviewer #1 indicates that in Fig. 1E there is fluorescent dirt, possibly dye remnants on the mCherry channel. We have included a confocal microscope image of another cleaner preparation clearly showing the separation of mCherry (CH1; excitation: 543 nm; emission: 572 ± 20 nm) and SiR700-lysosome channels (CH2; excitation: 635 nm; emission: 655-755). In this picture it is clearly shown that only a fraction of lysosomes is associated with the KFERQ-tagged fluorescent protein. First, myo-3p::KFERQ-PA-mCherry is expressed only in body wall muscle cells and not in pharyngeal muscle and nerve cells. Therefore, as observed in the new Fig 1E, lysosomes stained with the SiR700-lysosome probe in pharyngeal muscle and nerve cells do not correlate with mCherry signal (see Fig. 1E (d, h) ICA (intensity correlation analysis) image). Contrarily, a high intensity correlation between both channels is observed locally in most lysosomes in body wall muscle cells in the head area (see Fig. 1E (c, d, g, h) arrows) with some exceptions (Fig. 1E (c, d, g, h) arrowheads). Colocalization analysis was made locally in body wall muscle cells using the Intensity correlation analysis colocalization method, as shown in Supporting information S1 and described in more detail in materials and methods. The product of the differences from the mean (PDM) of each pixel intensity from each channel was spatially represented in pseudo colored ICA images with yellow indicating high positive values (high correlation) and blue indicating negative values (negative correlation or exclusion).

The merged and ICA images in Fig. 1E clearly show a high degree of colocalization only in lysosomes. Moreover, the absence of colocalization between the diffuse cytosolic PA-mCherry signal and the signal corresponding to the SiR700-lysosome fluorescence channel, suggests no signal bleed-through between the two channels. To corroborate the above statement, the fluorescence bleed-through controls between the different channels are shown. Supporting information Figure S1 now includes fluorescence channel crosstalk controls of worms expressing myo-3p::KFERQ-PA-mCherry and stained with the SiR700-lysosome probe. As shown in Fig S1A no fluorescence bleed-through of the SiR700-Lysosome probe in the mCherry channel (CH1; excitation: 543 nm; emission: 572 ± 20 nm) was detected in non-photoactivated worms. This is more evident in the CH1 image with enhanced contrast by histogram equalization (CH1 Equalized), where no spatial correlation of the fluorescence between both channels is observed. Since the SiR700 in vivo probe enters the worms through ingestion, there is a very strong fluorescence signal in the pharynx cavity. However, nor in this case neither in the intracellular punctate structures of head muscle cells observed in CH2 (CH2; excitation: 635 nm; emission: 655-755) are observed in the mCherry channel 1 (CH1). Similarly, in photoactivated myo-3p::KFERQ-PA-mCherry worms there is no substantial bleed-through of PA-mCherry fluorescence in SiR700-Lysosome fluorescence channel (CH2) as observed in Figure S1B.

5. Reviewer #1 indicates that most of the lysosomal marker is not absorbed in the PA-mCherry-expressing muscles cells of the head and that the two signals somehow anticorrelate at the whole cell level. In all our experiments we observed that the SiR700-Lysosome probe was properly absorbed in body wall muscle cells, however the number of lysosomes and the intensity of the lysosomal marker in pharyngeal cells was greater than in body wall muscle cells. In Fig 1E, we show that the SiR-700-lysosome probe was properly absorbed in KFERQ-PA-mCherry expressing cells. We provided single and merged fluorescent images in full frame figures and zoomed areas as required by reviewer #1.

S2 Fig. (A) The lysosomes are present in all tissues and therefore we observe green dots everywhere. The cherry marker (not KFERQ-PAmCherry) is expressed only in body wall muscle cells. This is a control experiment to show that that cherry and lysosomes (green) do not colocalize, while KFERQ-PAmCherry and lysosomes do. We explained how the colocalization studies were carried out in S1 and its figure legend.

6. Lines 302-304. There is a comment about this in the discussion line 444-456. Studies in human cells have previously demonstrated the existence of an interaction between macroautophagy and CMA [Kausin et al 20218], whereby cells respond to blockade of one of these pathways by up-regulating the other.

7. The goal of Fig. 4B is only to show that the expression of a-synuclein in body wall muscle cells affect the worms’ swimming behavior. Graph 4C compares the swimming behavior of the transgenic worms that express a-synuclein under feeding and starving conditions (when CMA is activated). It has been shown that starvation in WT worms decreases the rate of locomotion (Genetics, Vol. 216, 837–878 December 2020). Our point is to see the effect on α-synuclein.

8. The muscles in the head area are part of the body wall muscles. α-synuclein is expressed in all these muscle cells, as shown in Fig.4Ad. Fig.4Ac shows that the actin cytoskeleton of the body wall muscles is very disorganized. We perform the quantification of a-synuclein aggregates in the head region because it is a well-defined anatomic area.

9. True. We have added statistical tests in each figure legend.

Minor comments:

10. The reviewer is right. Line 69. “Specialized autophagy receptors” was changed to “Selective autophagy receptors”.

11. The word “triggers” was changed to “increases”.

12. Figure 1E was changed

13. Figures S1 was changed to

14. The word “dot” was changed for “puncta” throughout the text.

15. The mistake in line 140 was corrected.

16. line 175 syntaxis changed, protein-protein docking. "The lowest-energy conformer of the most populated cluster of each complex, which also had to be compatible with the steric hindrance of the membrane, was subjected to a 100 ns MD simulation using the same methods described for LMP-1 and LMP-2. RMSD and RMSF of HSP-1 in each simulation were later calculated using MDAnalysis."

17. line 186 We eliminated the workd “embryo”. The word “feed” was replaced with “fed”.

18. We did our preliminary experiments of photoactivation for 10 minutes. However, it was important to limit UV exposure, and we found that 5 minutes was enough to visualize KFERQ-PAmCherry. All experiments shown were done with 5-minute exposure time.

19. Line 210. The term “transfected” was changed for “transformed”.

20. Fig 1D legend. We added 1 asterisk for the p value p 0,0001.

21. Fig S1 and S2 were changed for provide a better explanation

22. The size points in graph Fig. 2B. were adjusted to the same size. There is statistically significant difference between starved control (RNAi) and starved *hsp-1* (RNAi): ****p< 0.0001. This was added in the graph Fig 2 B and F

23. We changed axes labelling for consistency in all figures.

24. There was a mistake. We fixed the figure with the appropriate pictures. Thank you very much!

25. We changed the graph to better show that there is in fact a statistical difference in the puncta number per head between starved *lmp-1* mutant and starved downregulated hsp-1 in the lmp-1 mutant background. Since hsp-1 is involved in CMA and in microautophagy, it is possible that there is increase macroautophagy due to the crosstalk between these pathways. (Kaushik S and Cuervo AM, 2018)

26. The conditions are different. For example, in Fig 2B the time after photoactivation is 4h until data collection. In Fig 3B, the time after photoactivation is only 2 h until data collection because we are looking at macroautophagy. For each experiment we monitor the same population at different times (of course different individuals from the same plates at different times), therefore there is internal consistency of the results.

27. We changed LC3 for “GABARAP”.

28. We added statistics in the figure legend.

29. Since there is no decrease in the number of puncta when *hsp-1* is knockdowned in the *lmp-1* mutant background (they are not additive), it means that they could be in the same pathway. Why is there a modest increment in the number of puncta? We could speculate that in the absence of both gene products there could be a compensatory macroautophagy mechanism as seen in mammals (Kaushik S and Cuervo AM. 2018. Mol. Cell Biol. 19, 365-381). It is also important to consider that this gene silencing strategy may not completely suppress the expression of hsp-1. According to this we modified the paragraph in the result section (now 297-301)

30. The reference was included in the text

31. We changed it for 40X.

Reviewer #2

The reviewer is right. What was originally submitted was a draft version of the figures. Therefore, they are of low quality. We have now uploaded the original eps figures, separated from the manuscript, where the puncta can be visualized. It is also important to consider that KFERQ-PAmCherry can always be observed in the cytosol. When the worms are starved, some of this marker is accumulated into the lysosomes. However, since KFERQ-PAmCherry is overexpressed in the muscle cells, much remains in the cytosol.

In the absence of LMP-1 (the KFERQ receptor), the number of puncta in the fed and staved conditions is similar, the is no change, as is the case in the WT (control RNAi). We see puncta under all conditions, including the fed condition. However, only when the receptor (LMP-1) is present we observed increased number of punta.

Figure 2E shows that WT and mutant worms have the same number of active lysosomes (SiR700-lysosome is based on the cathepsin D binding natural product pepstatin A). It is a control to show that the absence of LMP-1 does not affect lysosomal biogenesis and functionality.
