# Supplementary material for: KFERQ-selective protein autophagy in Caenorhabditis elegans depends on LMP-1
Source: PLoS One. 2025 Sep 2;20(9):e0330339. doi: 10.1371/journal.pone.0330339 (PMC12404552; doi:10.1371/journal.pone.0330339)
Supplement: Attachment — Submitted filename: Response to Reviewers july 2025.docx [file pone.0330339.s011.docx]

Answer to Reviewer

6. Review Comments to the Author

***Reviewer #1:*** *In this round of review authors have responded to all reviewers’ comments. There is now access to high resolution images and the representative masks (Fig. S1) are convincing. Apart from the point below I have no further comments.
Without experimental data, the response “What happens in the head muscles also occurs in the muscles of the rest of the body” is at the best case rather vague and misleading. Intestine is a separate tissue and its autofluorescence has characteristic pattern, it can be discriminated from muscle aggregates and the two tissues do not always align during imaging (both at xy as well as the z axis). The same applies for eggs. Even if they interfere with the smooth imaging of muscles cells, this should not affect the existence of aggregates. Imaging of aggregates in some of these cells that do not interfere with intestine or eggs should be feasible. Quantification would be ideal but at least the respective images are necessary.*

To answer the reviewer’s concern, we put together a series of pictures showing a-synuclein aggregates in muscle cells corresponding to the head, anterior mid-body, posterior mid-body, and tail region (Figure 1). Panel A, shows control animals and B, animals starved for 6 hours. The photographs on the left column were taken using an epifluorescence microscope adding some transmitted light, to visualize the pharynx in the head region, and other anatomical details in other parts of the body. Aggregates are seen in muscle cells in all regions of the worm’s body (head, anterior mid-body, posterior mid-body, and tail). Fewer aggregates are seen in the worms that were starved for 6 h (Panel B).

It is important to emphasize that our decision to quantify aggregates in the head region of individual worms, rather than in other areas, because this region can be readily and consistently delineated by the anatomical landmark of the pharynx, making quantification more reliable.

Moreover, *C. elegans* body wall muscle cells are similar to one another, arranged in four longitudinal bands and are all connected through gap junctions (*Front Physiol. 2014 Feb 11;5:40. doi: 10.3389/fphys.2014.00040. Gap junctions in C. elegans. Karina T Simonsen 1, Donald G Moerman 2, Christian C Naus 1*), from head to tail. All contribute to the worm’s sinusoidal crawling and swimming.

*Also, are the muscle cells in Fig. 4A located somewhere at the midbody (other than the head)? The images look somehow tilted. Please include the merge one and scale bar.*

The figure 4 A shows approximately the same mid-body region, but from different worms of the same strain. This approach was necessary due to technical incompatibilities between the phalloidin staining and GFP/YFP visualization. For phalloidin F-actin staining, worms must be frozen in liquid nitrogen, lyophilized, and treated with acetone (WormBook.org and Bio Protoc. 2021 Oct 5;11(19):e4183. doi: 10.21769/BioProtoc.4183). However, GFP fluorescence is abolished by acetone treatment, and GFP is also highly sensitive to dehydration. Therefore, aggregates were visualized *in vivo*. Scale bars were added.

**Figure 1**. Fluorescence microscopy images of muscle cells from control animals (A) and starved for 6 hours animals (B)The photographs on the left column were taken using an epifluorescence microscope adding some transmitted light, to visualize the pharynx in the head region, and other anatomical details in other parts of the body. Aggregates are seen in muscle cells in all regions of the worm’s body (head, anterior mid-body, posterior mid-body, and tail). Fewer aggregates are seen in the worms that were starved for 6 h (Panel B).

**Reviewer #2**: *This manuscript has some interesting findings. Though it has a huge limitation of quantitation of puncta/aggregates being subjective, it can be accepted due to the lack of alternative means to measure the puncta/aggregates*.
